# Hemoptysis, Causes and Treatment

**Published:** 1905-11

**Authors:** L. Sexton

**Affiliations:** Professor of Minor Surgery, Tulane University; Ex-President American Academy of Railway Surgeons, New Orleans, La.


For Texas Medical Journal.

### Hemoptysis, Causes and Treatment.

BY L. SEXTON, M. D.
Professor of Minor Surgery, Tulane University; Ex-President American Academy of Railway Surgeons, New Orleans, La.

Hemoptysis, being of sudden onset, it is not inappropriate to occasionally rehearse its causes and the newer remedies applied to its relief. While in the beginning tuberculosis usually makes itself known in this way, we need not hasten to the conclusion that phthisis is the only, though most common, cause of hemorrhage of the lungs.

Prominent among other conditions that bring about blood spitting, are pulmonary congestion caused by acute infectious diseases, as yellow fever, pneumonia, etc. Pulmonary embolism (infarction), ulceration of larynx or trachea form malignant disease or other cause. Emphysema and hard coughing resulting therefrom is another source of hemorrhage. Mitral disease of the heart, aneurysms, vicarious menstruation in amenorrhea, puerperal hemorrhagica, gouty heart in older persons, traumatism to the chest wall are among the most common causes of pulmonary hemorrhage. Severe muscular strain and mental excitement may occasionally result in blood spitting. These with other unmentioned causes are enough not to consign every case of broncho pulmonary hemorrhage to the tubercular class, even though 75 per cent of cases have that etiology.

### DIFFERENTIAL DIAGNOSIS.

*Hematemesis.*—Associated with gastric, splenic, or hepatic disease. Blood is dark; is vomited while coughing; acid reaction; mixed with food. Usually nausea and faintness precedes the hemorrhage. If from pharynx, is small in quantity and mixed with mucous. Examine nose and mouth for bleeding points. In ptyalism, odor and gums show the source.

*Hemoptysis.*—History points to either lung or heart disease, usually associated with cough. Red blood, and frothy, coughed-up small clots, if any; alkaline reaction; comes on suddenly. Salty taste in mouth. Tickling in throat.

*Prognosis.*—A hemorrhage for the time being helps the mucous membrane of a lung that is unduly congested, i. e., if all the blood is expectorated. If the hemorrhage from the upper lobe gravitates to the lower lobes, lobar inflammation or pneumonia may result. Niemeyer thought hemorrhage preceded, instead of followed the tubercular process. Lobar inflammation might convert a latent inflammation into an active process. Hemorrhage from the lungs or from any internal organ is always serious; when of tubercular origin it is especially so; when from a cavity, more than when due to a primary lesion.

Treatment resolves itself into quieting the tumultuous heart beat, rest in the recumbent position, and the cessation of speaking, coughing, or any lung exercise beyond the most simple respiratory acts. The patient should assume the reclining position when and wherever the hemorrhage begins, and should remain in this position, not speaking above a whisper till all evidence of recurrence has passed.

Diet should be cold and liquid in character, easily digested. Hot, stimulating food or drink should not be allowed. A well-filled ice bag should be placed over the heart and lungs with a fold of intervening flannel or soft cloth to make it more bearable and to absorb the moisture. This cold treatment is not suitable for old or very anemic cases. Agreeable saline purging to help deplete the pulmonary oedema is indicated. The one which will not upset the stomach is the best, for any effort at vomiting only dislodges clots and is liable to bring on another hemorrhage. Dry cupping over the chest is very helpful to the congested lungs.

One-fourth-grain doses of morphia hypodermically repeated often enough to prevent coughing and pain, to slow respiration, and quiet the patient is perhaps the most effectual remedy in the doctor's armamentarium.

In kidney trouble or where morphia is otherwise contraindicated, syrup of chloral or any agreeable preparation of the bromides may be given. In children, syrup of morphia and scillæ, equal parts, act well both in preventing coughing and keeping the patient quiet. Teaspoonful doses of the adrenalin solution, 1-1000, given with ice, or fluid extract ergot and syrup morphia, equal parts, teaspoonful doses, an hour apart, has controlled two cases successfully for me. The taking of plenty of ice is to be commended; the giving of salt, sugar of lead, gallic acid, does the stomach more harm than it does the hemorrhage good.

In prolonged hemorrhage from a pulmonary cavity 1-1000 of atropin sulphate has been used with a view of distributing the circulation to the peripheral extremities. In hemorrhage from mitral disease, ten-drop doses of tr. digitalis or strophanthus should be pushed. In gouty cases iodide of potash and gilsemium should be given as a constitutional measure. A convalescent patient should not inhale the fumes of tobacco or any irritating gases; should practice total abstinence. All bronchial troubles and tendency to cough should be given prompt attention. A sputum examination, if it reveals tubercle bacilli, would call for dry climate and the usual anti-tubercular treatment. The subsequent treatment of any case depends upon the cause.

